# Debridement, Antibiotic Pearls, and Retention of the Implant (DAPRI) in the Treatment of Early Periprosthetic Knee Joint Infections: A Literature Review

**DOI:** 10.3390/healthcare12080843

**Published:** 2024-04-16

**Authors:** Giovanni Vicenti, Elisa Pesare, Giulia Colasuonno, Claudio Buono, Federica Albano, Teresa Ladogana, Anna Claudia Passarelli, Giuseppe Solarino

**Affiliations:** Orthopaedics Unit, Department of Basic Medical Science, Neuroscience and Sensory Organs, School of Medicine, University of Bari “Aldo Moro”, AOU Consorziale Policlinico, 70124 Bari, Italy; dott.gvicenti@gmail.com (G.V.); g.colasuonno10@studenti.uniba.it (G.C.); c.buono6@studenti.uniba.it (C.B.); f.albano34@studenti.uniba.it (F.A.); t.ladogana@studenti.uniba.it (T.L.); a.passarelli2@studenti.uniba.it (A.C.P.); giuseppe.solarino@uniba.it (G.S.)

**Keywords:** DAPRI, early infection, knee, arthroplasty, calcium sulphate beads

## Abstract

(1) Background: Periprosthetic joint infections (PJIs) are severe and frightening complications in orthopaedic surgery, and they are generally divided into three categories: early infections (those occurring within the first 4–6 weeks), delayed infections (those occurring between 3 and 24 months), and late infections (those occurring more than 2 years after surgery). PJI treatment comprises “debridement, antibiotics, and implant retention” (DAIR), single-stage revision, and double-stage revision. Nowadays, to improve the chances of retaining an infected implant and to improve the traditional DAIR method, a modified surgical technique has been developed, named DAPRI (debridement, antibiotic pearls, and retention of the implant). Our study aims to present an up-to-date concept evaluation of the DAPRI technique and its success rate. (2) Methods: Preferred Reporting Items for Systematic Reviews and Meta-analyses (PRISMA) standards were followed, applying a protocol defined by the authors: a total of 765 articles were identified, and at the end of the screening process only 7 studies were included. (3) Results: Currently, the DAPRI procedure can be performed only on patients who have had PJI symptoms for less than 4 weeks, and in order to achieve the highest success rate, indications are quite strict: it is appropriate in patients with acute, superficial infections without sinus tract presence, and well-fixed implants with known sensitive bacteria. The DAPRI surgical method follows a step-by-step process consisting of a first phase of biofilm identification with intra-articular injection of methylene blue, followed by biofilm removal (thermic, mechanical, and chemical aggression), and a last step consisting of prevention of PJI recurrence by using calcium sulphate antibiotic-added beads. (4) Conclusions: The DAPRI approach improves the traditional DAIR technique. It is a correct treatment for acute and early haematogenous PJI, and improves the DAIR success rate.

## 1. Introduction

Early periprosthetic joint infection (PJI) is still a severe and frightening complication in orthopaedic surgery. Periprosthetic joint infection is one of the main causes of total knee arthroplasty (TKA) failure, along with instability and aseptic loosening of the implant [1]. PJI represents one of the principal causes of early revision of TKA, considering the devastating effect that the infection could have locally and systemically on the patient who could experience a worse quality of life [2]. A no less important consequence of PJI is the enormous impact on health spending: it significantly increases health costs [3]. As a matter of fact, considering this complication from a purely economic and managerial point of view, many authors have defined PJI as a real emergency in both the private and public welfare system. The infection rate following primary knee or hip replacement is estimated to be between 0.3 and 1.9% [4] and can greatly increase after revision surgery. When joint revision is required, this complication occurs in 39.6% of all surgical operations [5]. Specifically, periprosthetic joint infection (PJI) occurs in 1% of total hip arthroplasties (THAs) [6], in 1–2% of total knee arthroplasties (TKAs), and 0.1–0.8% of all cases of unicompartmental knee (UKA) arthroplasty. Furthermore, PJI has been found to occur in 13% of revision hip arthroplasties and 23% of revision knee arthroplasties [7].

Nonetheless, according to a recent systematic review, the average PJI rate of occurrence within the first six months after surgery was 4.2–4.8% [8]. PJI treatment comprises “debridement, antibiotics, and implant retention” (DAIR), single-stage revision, and double-stage revision, depending on different factors such as the timing of the diagnosis, microbiological agents, and patients’ general health status [9]. Every kind of implant-saving surgery depends on the timing of the PJI: signs of infection must have been present for less than 4 weeks (most favourable being less than seven days), and microorganism culture and identification are necessary [10].

PJIs are generally divided into three categories: early infections (those occurring within the first 4–6 weeks) are usually caused by highly virulent organisms; delayed infections (those occurring between 3 and 24 months) are usually caused by organisms of lower virulence; and last but not least, late infections (those occurring more than 2 years after surgery) are usually haematogenous in nature [11].

In late and chronic infections, there is indication of revision of the implant, which can be carried out in one or two stages depending on the identification of the microorganism, the degree of virulence, and the factors related to the host. In the case of an early infection or acute haematogenous infection (7 days from symptom onset), the most common treatment used is DAIR, which is considered a reasonable choice for patients who have a sinus tract-free and well-fixed prosthesis [12]. Compared to the other surgical treatment choices, the DAIR procedure is actually less aggressive and demanding in terms of invasiveness, difficulty of the technique, economic impact, morbidity, hospitalization, and bone stock preservation; however, it can be performed in few selected cases. DAIR’s first step consists in the debridement procedure of removing the haematoma, fibrous membranes, sinus tracts, and devitalized bone and soft tissues.

Nowadays, to improve the chances of retaining an infected implant and to improve the traditional DAIR method, a modified surgical technique has been developed, named DAPRI (debridement, antibiotic pearls, and retention of the implant) [13].

This technique aims to remove intra-articular biofilm on the implant surface and soft tissues through a combination of procedures: methylene blue staining, argon beam electrical stimulation, chlorhexidine gluconate brushing, and prolonged local antibiotic concentration. These techniques work in synergy and may lead to an increase in the overall success rate in implant-retention revision surgery.

It is well known that once biofilm is formed, bacteria become extremely resistant to any antibiotic; the use of chlorhexidine gluconate and an argon beam coagulator aid biofilm eradication by mechanical removal of microbes. Meanwhile, calcium sulphate antimicrobial-impregnated beads help to maintain ideal intra-articular antibiotic concentration through local action, providing prevention of systemic adverse effects commonly related to high levels of antibiotic administration [14].

The aim of this study is to present an up-to-date concept evaluation of the DAPRI technique and its success rate.

## 2. Materials and Methods

According to Preferred Reporting Items for Systematic Reviews and Meta-analyses (PRISMA) [15] guidelines, a comprehensive literature examination was conducted between January 2003 and December 2023 in the PubMed, Medline, Cochrane, and Google Scholar databases. The research was conducted using keywords such as “DAPRI”, “early infection”, “knee”, “arthroplasty”, and “Calcium Sulphate Beads”, along with Boolean operators AND and OR.

Two authors (P.E. and C.G.) collected evidence separately by searching several databases, generating a total of 765 articles. Levels II and III of the evidence were evaluated for inclusion criteria: reviews, systematic reviews, and meta-analyses focusing on infection in the orthopaedic field. We excluded in vitro or in vivo animal model studies and low-evidence research such as expert opinions, technical comments, and clinical trials.

The two authors previously mentioned screened the articles for the first time by reading the title of each paper included. Subsequently, they removed some publications through abstract review, excluding papers with exclusive abstracts available and papers that made no mention of DAPRI treatment.

After this phase, the data were extracted by another author (B.C.) and were checked for accuracy and completeness by a second member (A.F.). Information collected included the first author, title, authors, year of publication, study type, and study design.

Each included paper was then evaluated by full-text reading by two independent investigators (V.G. and B.C.). Discrepancies were resolved by consensus and, in cases of persistent disagreement during this phase, by consulting a third author who has extensive expertise in knee revision surgery (S.G.).

**Figure 1:**
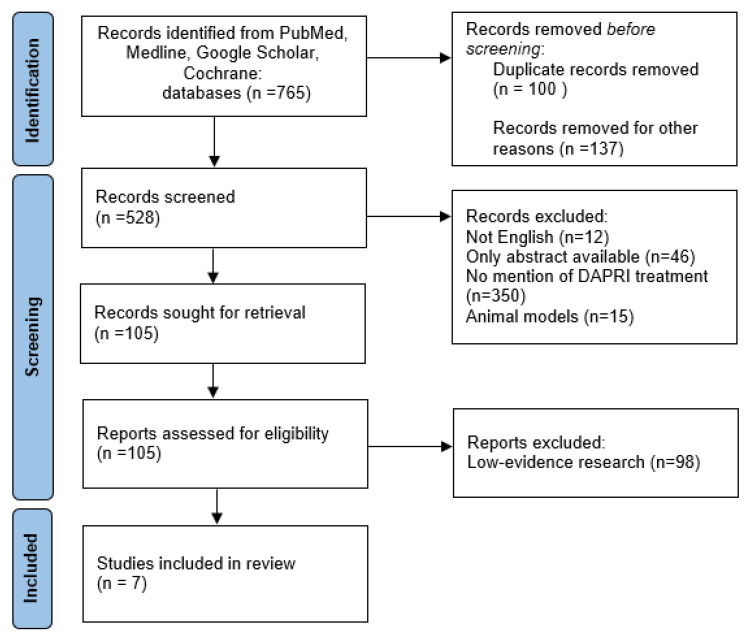
PRISMA flowchart.

As depicted in the study screening flowchart (Figure 1), the references of the examined studies were reviewed to identify grey literature and discover additional papers that may have been initially missed.

By reviewing the references of the already identified studies, we aimed to uncover additional sources that might provide valuable insights or perspectives related to our topic of interest. This approach helped us enhance the comprehensiveness of our literature review process and ensured that relevant information from a variety of sources was considered.

In the end, a total of 765 publications were found, and after selection using the inclusion criteria, a total of 7 studies were selected (Table 1).

## 3. Results

### 3.1. Periprosthetic Joint Infections (PJIs)

PJI is often linked to the necessity of multiple revision procedures, occurring in 23% of revision knee arthroplasties [7,17]. The implications of PJI extend to recurrent infections, prolonged courses of antibiotics, extended hospital stays, delayed aseptic loosening, and suboptimal functional outcomes [7]. Consequently, PJI places a substantial economic and logistical burden on the healthcare system and stands out as one of the most formidable complications, associated with elevated mortality and morbidity rates [18].

The ideal target of a PJI is to eradicate the infection and regain a pain-free, fully functional joint. There are several therapeutic methods available, but the most effective strategy remains a topic of debate.

Treatment options for periprosthetic joint infection encompass a range of approaches, including suppressive antibiotics, arthroscopic irrigation and debridement, open debridement with insert exchange, single-stage reimplantation, and two-stage reimplantation. The selection of the appropriate treatment strategy varies and is contingent on several key parameters. These parameters include implant integrity; the timing of the infection; host characteristics such as age, overall health, and immunologic state; the virulence of the infecting organism; and the individual needs of the patient. The choice of treatment is thus tailored to the specific circumstances of each case, ensuring the most effective and personalized approach for managing PJI.

### 3.2. PJI Definition

In 2013, over 400 experts of different medical branches with an interest in orthopaedic infections convened during the Infection Consensus Meeting [19] to reach a consensus on the management of PJIs.

According to their consensus, the definition of PJI can be established in the case of the identification of two positive periprosthetic cultures with phenotypically identical organisms.

Additionally, a PJI can be defined if a sinus tract communicating with the joint is present or if three of the minor criteria are met, which are as follows:

- Elevated serum C-reactive protein (CRP) and erythrocyte sedimentation rate (ESR) [20].
- Elevated synovial fluid white blood cell (WBC) count.
- Presence of a significant change on the leukocyte esterase test strip.
- Elevated synovial fluid polymorphonuclear neutrophil percentage (PMN%).
- Positive histological analysis of periprosthetic tissue.
- A single positive culture.

These criteria provide a comprehensive framework for diagnosing PJI, encompassing both microbiological and clinical indicators [19].

### 3.3. PJI Classification

The classification of periprosthetic joint infection (PJI) is critical for understanding the timing and type of the infection, as well as the most appropriate treatment. Diagnosis of PJI remains challenging, and current methods include a comprehensive assessment of serum and synovial biochemical [21] as well as microbiological data. Staphylococcus aureus, Propionibacterium acnes, Staphylococcus epidermidis, and coagulase-negative Staphylococcus are known as the most common bacteria responsible for most PJIs. However, it is noteworthy that the virulence of microorganisms varies concerning the timing of onset.

There are several classifications of PJI in the literature. According to Izakovicova et al. [22] in the latest EFORT society review, various types of PJI can be categorized by the time elapsed since the initial onset of symptoms. For perioperative cases, we define acute PJIs as those with symptom onset occurring within 4 weeks after surgery, while those presenting symptoms beyond 4 weeks after surgery are classified as delayed.

Tsukayama’s categorization system, created in the 1990s, is still actual, offering a comprehensive perspective on PJIs by dividing them into four distinct types instead [23].

This categorization takes into consideration both the time since the surgery and the presumed process of infection, including positive intraoperative cultures, early postoperative infections, and late haematogenous chronic infections [7].

Nowadays, PJIs are divided into three types:

- “Early Infections” are those infections that develop within the first 4 weeks postoperatively. Typically, they are associated with virulent organisms, such as Staphylococcus aureus and certain Gram-negative bacilli. These infections often cause an elevated erythrocyte sedimentation rate (ESR), joint pain, swelling, redness, warmth at the site of the implant, and fever [24]. These infections are characterized by immature biofilm formation [22].
- “Delayed Infections” are those infections occurring more than 4 weeks postoperatively. The timeframe for delayed infections extends beyond the immediate postoperative period, suggesting a more subacute or insidious onset. They are usually caused by less virulent species than early infections. Staphylococci or Cutibacterium acnes are some of the most common microorganisms responsible. People affected do not have clear symptoms, but they complain of persistent bone pain, swelling, and signs of systemic inflammation. Radiographies can show signs of implant loosening [7]. These infections are characterized by mature biofilm formation [22].
- ”Late Infections” are those infections occurring more than 24 months postoperatively [7]. They are characterized by a prolonged timeframe, indicating a delayed onset well after the initial surgical intervention. It has been shown that Staphylococci can cause late haematogenous PJIs, not only the early-onset ones [7]. Symptoms may resemble those of delayed infections, with a potential for chronicity and progressive joint deterioration. Late infections such as delayed ones may present with a more indolent course, necessitating a careful and thorough diagnostic approach to differentiate them from other potential causes of postoperative joint symptoms [7].

### 3.4. Indications and Contraindications: DAPRI and DAIR

It is now widely known that PJI consists of four different categories divided by postoperative onset time. Two out of the four are the most common types of PJI, which were classified and described by Tsukayama et al.: type IIb, early deep postoperative infection (which develops within 4 weeks after surgery), and type III, acute haematogenous infection [7,23,25]. Recognizing and categorizing PJIs according to their type is crucial for tailoring effective and targeted treatment plans that address the specific characteristics and challenges associated with each infection category.

The type of surgical treatment is traditionally linked to the timing: in chronic and late infections, implant revision is recommended, and it can be performed in one or two stages depending on the identification of the pathogen, its degree of virulence, and host-related factors [26,27]. The DAIR protocol is frequently used to preserve the implant in cases of acute infection (within 4–6 weeks after surgery) or early haematogenic (within 7 days of the symptom’s onset). The aim of this less disruptive surgical technique is to preserve a functional implant and avoid the significant morbidity of implant removal and subsequent surgical procedures. The indications and contraindications of using debridement, antibiotics, and implant retention have been discussed in the Hip and Knee section of ICM 2018. The patients who are considered targets for DAIR procedures are the ones diagnosed with a periprosthetic joint infection without the loosening of the implant. This is the main indication for this surgical technique [12,28,29]. Concerning contraindications, there are no absolute contraindications to perform a DAIR procedure, but it is strongly recommended not to perform this technique in cases of high failure rate caused by prosthesis retention. A diagnosis of chronic periprosthetic joint infection (PJI) can be considered an absolute contraindication to the DAIR procedure. In this case, the DAIR procedure represents the wrong choice of treatment because in chronic infection the process of formation and maturation of the biofilm, thanks to the presence of “persister cells”, is complete. For this reason, it is impossible to eradicate the infection by keeping the prosthesis in place, and DAIR surgical steps are not recommended.

Despite this, different authors have demonstrated the efficacy in 50% of cases of DAIR treatment for PJI with a duration of symptoms exceeding one month: they showed an infection eradication rate of 50% to 80%, but it is important to underline that this happens only when the technique is used appropriately in selected patients [12]. Given that DAIR is the surgical procedure of choice in selected patients who have early acute infection (within 4 weeks of surgery) and/or acute haematogenous infection, it can be considered an urgent but not emergency type of treatment. The time between the diagnosis of infection and the execution of the DAIR procedure is an important predictive factor for the success of the technique and the eradication of the infection itself [30]. A number of factors influence the success rates of DAIR such as the type of infecting microorganism, duration of symptoms, length of antibiotic therapy, and latency period between the development of symptoms and the time of surgery [31,32]. According to ICM 2018, no set time limit after which DAIR should not be attempted has been defined. Nonetheless, symptoms lasting less than one week have been linked to a higher success rate. Furthermore, the age of the implant has been identified as a prognostic factor for successful DAIR treatment [8,30,33,34].

Microbiological diagnosis is crucial for infection confirmation as well as evaluating the antimicrobial susceptibility of the pathogen(s) to guide antimicrobial therapy. The determination of the infecting pathogen is desirable, and it could encourage or discourage the execution of DAIR. Despite this, the debate is whether waiting to determine the infective organism would adversely affect the outcome of DAIR and a timely intervention [35].

Regarding the infecting pathogen’s variable, in the “Hip and Knee Section, Treatment, Debridement and Retention of Implant: Proceedings of International Consensus on Orthopaedic Infections”, for each bacterium of the pathogens identified to be most commonly responsible for PJI, the rate of success of the DAIR procedure has been reported as follows: Staphylococcus aureus (13–90%), Gram-negative bacilli (27–94%), and Streptococci (40–94%). In particular, accurate success rates of *S. aureus*, streptococci, enterococci, and Gram-negative bacilli were identified, which were 55%, 58%, 51%, and 68%, respectively.

Recently, as previously mentioned, DAPRI has been introduced as a different approach created to improve the success rate of traditional DAIR surgery [8,32,33].

According to the 2018 ICM definition [36] of acute infection, the current authors performed DAPRI only on patients who showed PJI symptoms, excluding sinus tract presence [26], for less than 4 weeks from the presentation to the surgical team. 

In order to achieve the highest success rate, indications are quite strict [37]: it is appropriate in patients with acute, superficial infections and well-fixed implants with known sensitive bacteria [30,38,39].

### 3.5. DAPRI Procedure

Before performing DAPRI, preoperative therapy with antibiotics is not given to improve the sensitivity of intraoperative cultures, five of which are typically collected to obtain microbiological isolation. An arthrocentesis is performed, and the synovial fluid obtained is sent to the laboratory for culture examination [40].

The DAPRI surgical method follows a step-by-step process:

- Biofilm identification.

Prior to the beginning of the surgical procedure, an injection of 50 cc of diluted (0.1%) methylene blue (40 cc of saline and 10 cc of 0.5% methylene blue solution) is carried out in order to enhance the biofilm that can be easily recognizable after capsulotomy [9].

This step is an evolution of the technique described by Shaw in 2017 which used the methylene blue after performing the arthrotomy in order to be sure of the spread of the solution. The intra-articular injection of methylene blue performed before the arthrotomy is a crucial step that improves the technique because it avoids external leaking. After this injection, the spread of the solution in the knee is obtained by multiple rounds of flexion and extension of the knee for at least 1 min [10]. Methylene blue is known to stain bacterial biofilm. To prevent methylene blue from leaking into the surrounding tissues, under sterile settings, arthrocentesis is conducted before arthrotomy is carried out [10].

As previously mentioned, methylene blue is drained as much as possible from the joint, and then the remaining colour is aspirated from the joint following arthrotomy.

Following a standard medial parapatellar approach and the capsulotomy, the blue staining of all intra-articular surfaces can be identified.

- Biofilm removal.

**Figure 2:**
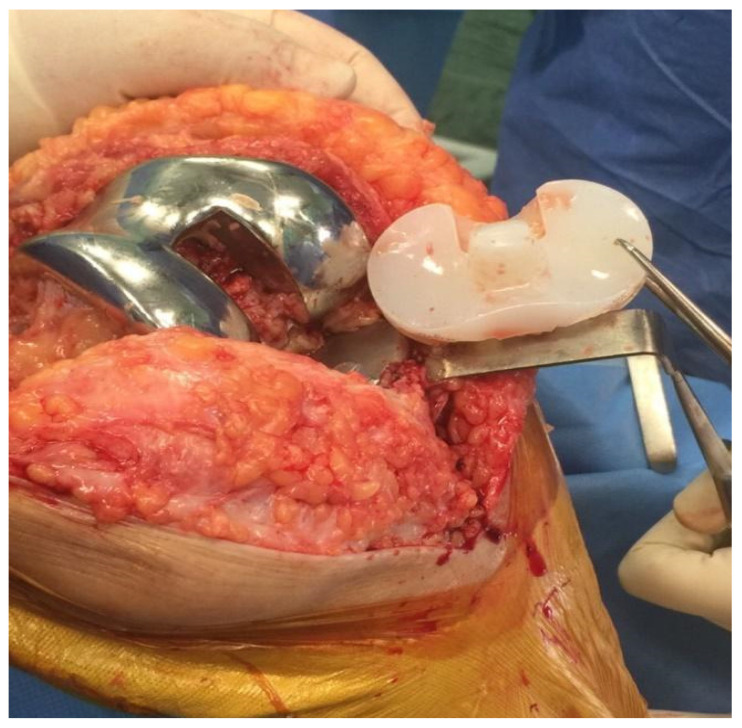
Polyethylene liner removal phase.

After a generous skin incision, the first step consists of polyethylene liner removal and, if possible, analysing microbiological contamination through the sonication process, but this is not mandatory [27] (Figure 2).

Then, aggressive and radical “tumour-like” synovectomy will help eliminate any infected soft-tissue biofilm formation.

Electrical stimulation has been demonstrated to detach biofilm from implant surfaces, although the eventual compromission of the structural proprieties of the implants is still controversial.

The highlighted soft-tissue biofilm is removed using electrocautery, and an aggressive tumour-like synovectomy is performed. After that, the biofilm can be removed by three methods of aggression: thermic, mechanical, and chemical [16].

The thermally guided removal is performed after the polyethylene insert removal from the prosthesis. This consists of using an argon beam coagulator in a painting, brush-like fashion on all visible surfaces on the femoral and tibial components [9]. The efficacy of these techniques is due to the fact that an electrical stimulation can facilitate the detachment of biofilm from the orthopaedic implant surface. The use of an argon beam coagulator damages biofilm stability.

After that, the mechanical and chemical removal of the biofilm is performed by scrubbing all visible implant surface components using a 2% chlorhexidine gluconate-impregnated brush [13].

At the very end of this procedure’s step, a generous application of an acetic acid, benzalkonium chloride (BZK)-based surgical lavage solution (Bactisure, Zimmer–Biomet, Warsaw, IN, USA), as an antimicrobial solution is performed [41]. After its application, an abundant pulse lavage with 9 L of povidone–iodine added to saline is always performed in order to dilute and thus reduce the local toxicity of the solution previously applied [41].

- Prevention of PJI recurrence.

Once the intra-articular space is believed to be free from biofilm presence, the wound is provisionally closed in order to prepare a new sterile surgical field. It is crucial at this point of the whole surgery that the surgical team scrubs out and then re-enters the operating room after changing their surgical gloves in order to ensure sterility, which the procedure demands at this time. After wound re-opening, further abundant irrigation of the joint has to be performed using saline pulse irrigation. After that, it is possible to reimplant the new modular prosthesis components. 

There is a lack of consensus about the irrigation procedure: in the past years, some surgeons utilized an acetic acid, benzalkonium chloride (BZK)-based surgical lavage solution (Bactisure, Zimmer–Biomet, Warsaw, IN, USA) added as an antimicrobial solution [16].

Nowadays, according to some authors, the best choice is pulsed irrigation in order to mechanically remove the biofilm. Indelli et al. [10] and Ghirardelli et al. [9] used pulse irrigation with 9 L (L) of povidone–iodine added to saline [9,10]. Calanna et al. [13] preferred pulse irrigation with 9 L of bacitracin added to saline.

The ideal irrigation solution has not been determined due to the absence of clinical research evaluating the efficacy of different antiseptic solutions and comparing antiseptic versus no antiseptic irrigation methods [42].

**Figure 3:**
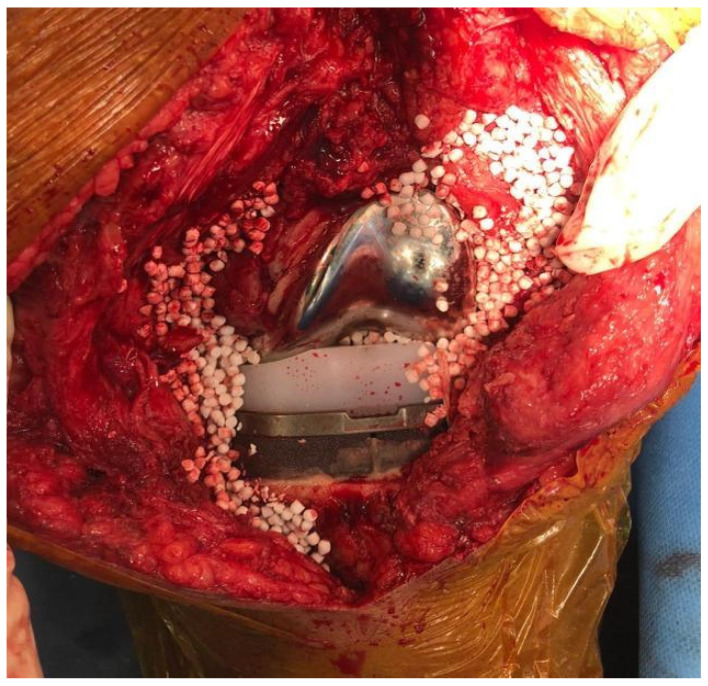
Calcium sulphate antibiotic-added beads.

Before the wound closure, 10 cc of calcium sulphate antibiotic-added beads (Stimulan, Biocomposites, Keele, UK) previously prepared on the back table are placed in the joint on the prosthesis components and in the near soft tissue [14]. A 10 mL kit of PG-CSH (Stimulan; Biocomposites Ltd., Keele, UK) is combined with 1 g of vancomycin powder, 0.8 g of tobramycin, and, according to the antibiogram or to the preoperative molecular testing result obtained at the time of microorganism identification, a third antibiotic added to the bead paste [14] (Figure 3).

The paste obtained by mixing the components mentioned above is then compressed in a 3 mm and 4.8 mm diameter hemispherical pattern-shaped flexible mould.

The beads provide a biocompatible and absorbable intra-articular delivery technology, allowing for continuous local release and longer local persistence of the specific antibiotic. The beads help to preserve the local antibiotic concentration for over a month [43].

The calcium sulphate beads are normally reabsorbed after approximately 6 weeks, as compared to PMMA (polymethylmethacrylate) spheres, which must be removed and might function as a substrate for subsequent bacterial colonization [16].

The most common complications of beads, as mentioned in the literature, are wound problems caused by exudate development and heterotopic ossifications.

Soft tissues can be closed in a standard fashion, making sure to seal the joint capsule in order to avoid postoperative drainage, which is a well-known complication of the use of calcium sulphate beads [13].

As the components are retained, a postoperative antibiogram-based antibiotic therapy, recommended by infectious disease specialists, has to be carried out for 12 weeks: 6 weeks of intravenous treatment, followed by 6 weeks of oral antibiotic subministration [10].

## 4. Conclusions

The DAPRI approach represents a notable advancement beyond the traditional DAIR technique. By incorporating intra-articular methylene blue, an argon beam coagulator, and chlorhexidine gluconate, it effectively addresses the challenge of biofilm elimination. Additionally, the integration of calcium sulphate antibiotic beads extends the concentration of antibiotics within the joint.

Positioned as a suitable and effective treatment for acute and early haematogenous PJI, the DAPRI technique enhances the success rate of the DAIR procedure. This innovative approach offers a promising strategy for managing infections, particularly those with biofilm presence. Achieving the highest success rate is contingent upon strict adherence to the specific indications of the DAPRI procedure. This targeted application ensures optimal outcomes by addressing clinical scenarios where the approach is most likely to be effective.

While the DAPRI technique shows promise, further studies are essential to enhance our understanding of its application and identify the precise clinical contexts in which it can be most beneficial. Continued research will contribute to refining the knowledge surrounding this procedure and aid in determining its optimal application fields.

## Figures and Tables

**Table 1:** Table of Included Study Details.

Author Name	Title	Type of Article	Year
Reinisch et al. [6]	Local antibiotic treatment with calcium sulphate as carrier material improves the outcome of debridement, antibiotics, and implant retention procedures for periprosthetic joint infections after hip arthroplasty—A retrospective study	Retrospective study	2022
Ghirardelli et al. [9]	Debridement, antibiotic pearls, and retention of the implant in the treatment of infected total hip arthroplasty	Editorial	2020
Indelli et al. [10]	Debridement, Antibiotic Pearls, and Retention of the Implant	Article and Review	2023
	(DAPRI) in the Treatment of Early Periprosthetic Joint		
	Infections: A Consecutive Series		
Calanna et al. [13]	Debridement, antibiotic pearls, and retention of the implant (DAPRI): A modified technique for implant retention in total knee arthroplasty PJI treatment	Review	2019
Abosala et al. [14]	The Use of Calcium Sulphate beads in Periprosthetic	Systematic Review	2020
	Joint Infection, a systematic review		
Tarar et al. [16]	Wound Leakage with the Use of Calcium Sulphate Beads in Prosthetic Joint Surgeries: A Systematic Review	Systematic Review	2021

## Data Availability

Data contained within the article.
